# Impact of selective LDL apheresis on serum chemerin levels in patients with hypercholesterolemia

**DOI:** 10.1186/s12944-016-0353-x

**Published:** 2016-10-18

**Authors:** Viktória E. Varga, Hajnalka Lőrincz, Noémi Zsíros, Péter Fülöp, Ildikó Seres, György Paragh, József Balla, Mariann Harangi

**Affiliations:** Department of Internal Medicine, Faculty of Medicine, University of Debrecen, Nagyerdei krt. 98, H-4032 Debrecen, Hungary

**Keywords:** Familial hypercholesterolemia, Chemerin, LDL subfractions, HDL subfractions, LDL apheresis

## Abstract

**Background:**

Selective low-density lipoprotein (LDL) apheresis is commonly used to treat patients with familial hypercholesterolemia (FH). Chemerin is an adipokine with putative roles in the regulation of lipid metabolism.

**Methods:**

In our pilot study, we measured serum chemerin levels by enzyme-linked immunosorbent assay in six severe heterozygous FH patients before and after their first LDL apheresis treatments using the technique of direct adsorption of lipoproteins (DALI).

**Results:**

The first treatment sessions decreased serum chemerin levels by an average of 27.26 %. While following one patient, 12 months of regular LDL apheresis resulted in a permanent reduction in his serum chemerin level. Changes in the lipoprotein subfractions measured by gel electrophoresis (Lipoprint) correlated with the reduction of chemerin levels. Furthermore, we eluted and then measured chemerin bound to the DALI column.

**Conclusion:**

We conclude that LDL apheresis decreases the circulating level of chemerin by binding the protein to the column and thus improves lipoprotein subfraction pattern.

## Background

Familial hypercholesterolemia (FH) is an autosomal dominant disorder caused most commonly by mutations in the low-density lipoprotein (LDL) receptor gene leading to extremely high plasma LDL-cholesterol (LDL-C) levels and increased risk of premature cardiovascular disease [1]. Mutations in the genes encoding either apolipoprotein (Apo)B100, proprotein convertase subtilisin/kexin type 9 (PCSK9) or autosomal recessive hypercholesterolemia adaptor protein also cause FH phenotype [2]. Although having lower LDL-C and total cholesterol levels compared to homozygous FH patients, heterozygotes still possess a significantly higher risk of cardiovascular diseases they are left untreated [3]. Homozygous FH patients are usually resistant to routine lipid lowering therapy and many heterozygous FH patients also do not respond adequately to these medications.

Selective LDL apheresis is an extracorporeal lipid lowering therapy which significantly can reduce the levels of ApoB-containing particles including LDL, Lipoprotein(a) (Lp(a)) and very-low-density lipoprotein (VLDL). Therefore, LDL apheresis is indicated to treat hyperlipidemia both in homozygous and severe heterozygous FH patients. Besides lipid lowering, several other beneficial effects of LDL apheresis have been reported including anti-atherogenic, anti-thrombotic and anti-inflammatory actions. As reviewed elsewhere, these lipid lowering therapies are also reported to reduce the levels of several pro-inflammatory peptides and cytokines [4].

Secreted from the white adipose tissue, adipo(cyto)kines play a crucial role in the regulation of lipid and glucose metabolism. To date, only a few studies examined the impact of LDL apheresis on adipokine levels. Leitner et al. reported that serum leptin levels decreased by 42 % after 14 months in a patient on regular LDL apheresis leading to ravenous hunger and weight gain [5], while Krautbauer et al. found no changes in serum adiponectin levels after LDL apheresis treatment [6]. Resistin and adiponectin levels were also found to be unaffected by LDL apheresis in another investigation [7]. Until now, there is no data on the impact of LDL-apheresis on the serum levels of chemerin.

Chemerin is mainly expressed in white adipose tissue, liver, kidney, skin and intestine [8], It regulates adipogenesis, adipocyte metabolism and lipolysis and controls adipocyte differentiation as well [9]. Its receptor is also expressed on different inflammatory cells including macrophages, immature dendritic cells and natural killer cells, inducing their migration to the site of inflammation [10]. Furthermore, circulating chemerin was reported to participate in the regulation of lipid and glucose metabolism [11]. Therefore, chemerin is considered to be associated with the development of obesity, insulin resistance and metabolic syndrome [12]. Although an anti-inflammatory effect of chemerin was also described [13, 14], there is a growing evidence that chemerin acts as a pro-inflammatory peptide [15]. Hence, chemerin may play a role in atherogenesis.

Previously, we found significant correlations between serum chemerin levels and lipoprotein subfractions in nondiabetic obese patients [16]. Circulating chemerin concentrations correlated positively with LDL-C levels, while an inverse correlation was found between chemerin and high-density lipoprotein- (HDL)-cholesterol (HDL-C) levels. Furthermore, significant positive correlations were detected between serum chemerin levels and small dense LDL, intermediate and small HDL subfractions, respectively. On the other hand, negative correlations were observed between chemerin levels and mean LDL size and large HDL subfraction, respectively. Based on these results, we concluded that chemerin might have an unfavorable effect on lipoprotein metabolism.

We hypothesized that LDL apheresis treatment may decrease serum chemerin levels that may enhance the beneficial effect of LDL apheresis on lipid parameters. Therefore, the aim of this study was to investigate the short- and long-term effects of selective LDL-apheresis on serum levels of chemerin in patients with severe heterozygous FH. We also measured the chemerin binding capacity of the columns used for LDL apheresis.

## Methods

### Patients

We enrolled six patients (three females and three males) with severe heterozygous FH before their first selective LDL apheresis treatment.

Characteristics and laboratory parameters before LDL apheresis are shown in Table 1.

**Table 1:** Patient characteristics and lipid parameters before and after LDL apheresis

	Before treatment	After treatment	Change in %
Age (years)	60.57 ± 4.79		
Gender (female/male)	3/3		
Body mass index (kg/m^2^)	26.19 ± 2.14		
Waist circumference (cm)	101 ± 8.19		
Total cholesterol (mmol/L)	9.25 ± 1.49	4.41 ± 0.99***	−52.58
LDL-C (mmol/L)	6.92 ± 1.34	2.69 ± 0.97***	−61.84
HDL-C (mmol/L)	1.40 ± 0.31	1.24 ± 0.16*	−10.46
Apo B (g/L)	2.10 ± 0.42	0.82 ± 0.28***	−61.49
Apo A (g/L)	1.69 ± 0.19	1.44 ± 0.10**	−14.09
Creatinine (μmol/L)	94.33 ± 58.18	82.33 ± 52.95**	−13.52
Serum chemerin (ng/mL)	82.34 ± 36.30	59.09 ± 24.89**	−27.26
Large LDL (%)	27.36 ± 6.64	23.72 ± 4.62	−12.20
Small-dense LDL (%)	4.86 ± 4.27	1.14 ± 1.57**	−83.81
Mean LDL size (nm)	26.64 ± 0.568	26.98 ± 0.402*	0.129
Large HDL (%)	21.08 ± 4.79	24.36 ± 4.25	16.70
Intermediate HDL (%)	45.16 ± 5.25	50.02 ± 3.47	11.45
Small HDL (%)	33.76 ± 8.66	25.62 ± 5.75**	−23.44

Data are presented as mean ± standard deviation (SD). *p < 0.05; **p < 0.01; ***p < 0.001

### LDL apheresis

LDL apheresis was carried out by direct adsorption of lipoproteins (DALI) (Fresenius GmbH, Germany), the DALI 750 adsorber was incorporated in the extracorporeal circuit. DALI primer solution, acid citrate dextrose formula A (ACD-A) solution, blood lines, and hemadsorption monitor 4008 ADS (Fresenius HemoCare Adsorber Technology GmbH, St. Wendel, Germany) were used. Prior to the session, the adsorbers were rinsed with 3 *×* 2000 mL of primer solution at a flow rate of 400 mL/min. The first 2 L contained 20000 IU of heparin. The adsorbers were saturated with citrate during priming. The patient received ACD-A infusion during the session. ACD-A was first mixed with the patient’s blood at a ratio of 1:20, which was reduced to 1:40 after 1500 mL of blood were treated. Two bilateral vascular accesses by vein puncture in the median cubital veins were established. At the start of the session, the patient was only connected to the afferent (arterial) line of the extracorporeal circuit. All sessions were carried out under blood pressure monitoring. Venous blood samples were drawn at the start and at the end of each session.

### Sample collection and laboratory measurements

Serum samples were separated by centrifugation at 4 °C at 3500 *g* for 10 min. The routine laboratory parameters were determined from fresh sera with Cobas c501 analyzer (Roche Ltd. Mannheim, Germany). Total cholesterol levels were measured by using enzymatic, colorimetric tests (cholesterol oxidase-p-aminophenazone – GPOD-PAP; Modular P-800 analyzer; Roche/Hitachi). HDL cholesterol and LDL cholesterol levels were determined by a homogenous enzymatic, colorimetric assay (Roche HDL-C plus 3rd generation and Roche LDL-C plus 2nd generation, respectively). Apo A-I, ApoB and Lp(a) examinations were performed by immunoturbidimetric assays (Tina-quant apolipoprotein A-I ver. 2, Tina-quant apolipoprotein B ver. 2 and Tina-quant lipoprotein(a) ver. 2, respectively). The tests were performed according to the recommendation of the manufacturer. Sera were kept frozen at −70 °C for subsequent lipoprotein subfraction analysis and ELISA measurements.

### Chemerin elution

We eluted chemerin from the apheresis column on the basis of the modified method of Dihazi et al. [17]. At the end of the session, DALI 750 columns were washed with phosphate-buffered saline (PBS) buffer of pH 7.4. Protein elution was carried out in a three-step elution process using 250 mL each of three acetate buffer solutions of different pH, in the following order: pH 5.0, pH 4.0, pH 3.0.

### LDL subfraction analysis

LDL subfractions were detected by an electrophoretic method on polyacrylamide gel using the Lipoprint System (Quantimetrix Corp., Redondo Beach, CA, USA), according to the manufacturer’s instructions. 25 μL of serum samples were added to polyacrylamide gel tubes along with 200 μL a loading gel solution containing Sudan Black as a lipophilic dye. The sample loading gel mixture was photopolymerized for 30 min at room temperature prior to electrophoresis at a constant of 3 mA/tube for one hour. Each electrophoresis chamber contained a quality control, which was provided by the manufacturer (Liposure Serum Lipoprotein Control; Quantimetrix Corp., Redondo Beach, CA, USA). For quantification, scanning was performed with an ArtixScan M1 digital scanner (Microtek International Inc., Santa Fe Springs, CA). Lipoprotein fractions (bands) were identified after electrophoresis by their mobility (Rf) using VLDL as the reference point (Rf 0.0) and HDL as the ending reference point (Rf 1.0). In between, LDL subfractions were distributed starting from LDL 1 to LDL7 (Rf 0.32, 0.38, 0.45, 0.51, 0.56, 0.6 and 0.64, respectively). LDL1 and LDL2 bands correspond to large buoyant LDL subclasses, whereas LDL3-7 bands correspond to small dense LDL subclasses. The percentages of the area under the curve (AUC%) for the VLDL, intermediate-density lipoprotein (IDL), LDL (maximum 7 subfractions), and HDL peaks were calculated with Lipoware computer software (Quantimetrix Corp., Redondo Beach, CA, USA). Proportion of large LDL (large LDL %) was defined as the sum of the percentage of LDL1 and LDL2, whereas proportion of small LDL (small LDL %) was defined as the sum of LDL3- LDL7. Mean LDL size (nm) was also calculated by the Lipoware software.

### HDL subfraction analysis

High density lipoprotein subfractions were also detected by Lipoprint, according to the manufacturer’s instructions. Briefly, 25 μL of serum sample was added to polyacrylamide gel tubes along with 300 μL of a loading gel solution containing Sudan Black as a lipophilic dye. After 30 min of photopolimerization at room temperature, electrophoresis was performed for 50 min with 3 mA/tube. Each electrophoresis chamber contained a quality control, which provided by the same manufacturer. Stained HDL subfractions (bands) were identified by their electrophoretic mobility. LDL/VLDL band was used as the starting reference point (Rf 0.0), while albumin served as the ending reference point (Rf 1.0). AUC% was calculated with the Lipoware computer software. Distributed between the reference points, HDL subfractions were grouped into three major classes: large (HDL1-HDL3), intermediate (HDL4-HDL7) and small (HDL8-HDL10) HDL subfractions.

### Serum chemerin concentration measurement

Serum chemerin concentrations were measured by a commercially available ELISA kit (Human Chemerin Quantikine ELISA, cat. number: DCHM00, R&D Systems, MN, USA), according to the recommendations of the manufacturer. The intra- and interassay coefficients of variations were <10 % and <12 %, respectively.

### Determination of chemerin concentration in eluted solution

Chemerin concentrations of the eluted solutions were measured by a commercially available ELISA kit (Human Chemerin DuoSet ELISA, cat. number: DY2324, R&D Systems, MN, USA).

### Statistical methods

Statistical analyses were performed by STATISTICA (version 6.0) (Statsoft Inc., Tulsa, OK, USA) and IBM Statistical Package for the Social Sciences (SPSS) Statistics (version 19) (IBM Corp., Armonk, NY, USA) computer softwares. Normality of distribution was tested by Kolmogorov-Smirnov test and data were analyzed with paired *t* test. Data were expressed as means ± standard deviation (SD) or median (upper quartile-lower quartile). A value of *P* < 0.05 was considered to be statistically significant. Friedman-ANOVA and Kendall Coeff. of concordance test was used to compare eluted chemerin concentrations using solution buffers of various pH.

## Results

**Fig. 1:**
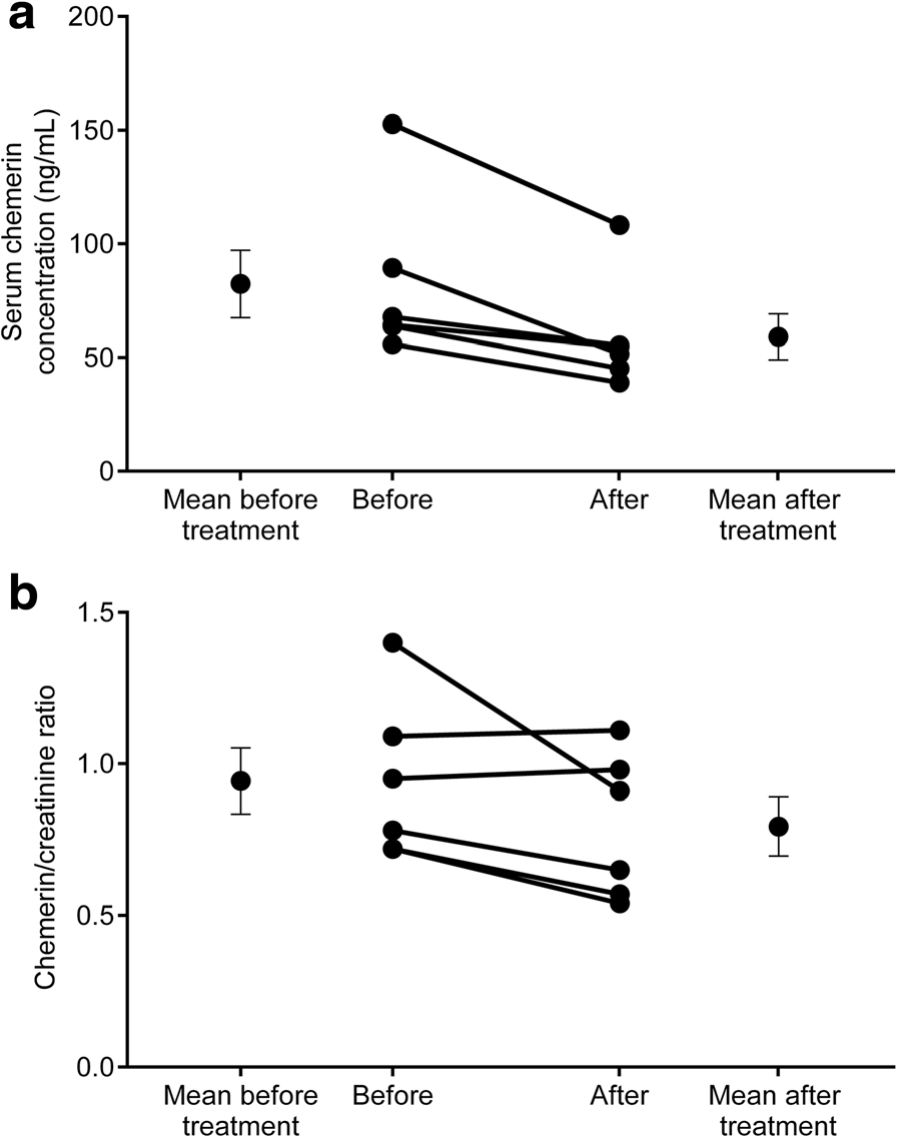
**a** Serum chemerin levels of the six patients (P1-P6) in ng/mL and; **b** Serum chemerin/creatinine ratios of the six patients (P1-P6) in % immediately before and after their first selective LDL apheresis sessions using DALI 750 columns. Abbreviation: SEM, standard error of the mean

Anthropometric and laboratory characteristics of the study participants are summarized in Table 1. After the first LDL apheresis, serum chemerin levels significantly decreased in all patients, on the average by 27.26 % (from the mean of 82.34 ng/mL to 59.09 ng/mL). To exclude the effect of dilution caused by the treatment we also calculated the serum chemerin/creatinine ratios, which also showed a significant decrease of 16.65 % after the first sessions. Reductions in individual serum chemerin levels and chemerin/creatinine ratios are shown in Fig. 1.

**Fig. 2:**
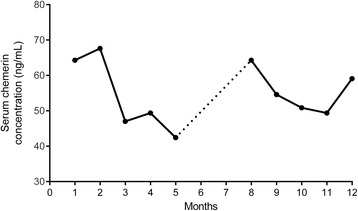
Changes in serum chemerin levels of a patient (P2) measured before LDL apheresis sessions during a 12-month period. Treatment has been suspended for three months (labelled with dotted line)

In order to examine the long-term effect of LDL apheresis on serum chemerin concentrations, we followed the serum chemerin levels of patient 2 (P2, selected randomly) before and after 10 LDL apheresis sessions. We found that chemerin levels showed a decreasing tendency during this 12-month follow-up. It has to be mentioned that the patient asked to suspend the apheresis for 3 months after the fifth session. After this period, we observed a marked increase in the circulating level reaching its initial value. Notably, serum chemerin concentration started to decrease after restarting LDL apheresis, indicating the importance of continuous treatment to maintain permanent reduction of serum chemerin levels (Fig. 2).

To prove that chemerin physically binds to the column, we eluted protein fractions from the apheresis column with three different acetate buffer solutions of different pH. The chemerin concentrations of the different solutions are shown in Table 2. Significantly more protein was eluted by buffer solutions of higher pH (*p* < 0.01).

**Table 2:** Chemerin levels of solution eluted from the LDL apheresis column in ng/mL

	Patient 1	Patient 2	Patient 3	Patient 4	Patient 5	Patient 6	Average
pH 3.0	148.89	79.73	71.69	134.69	37.82	24.77	75.7 (37.8–134.7)
pH 4.0	383.28	187.44	169.43	112.59	122.23	124.62	147.0 (122.2–187.4)
pH 5.0	649.73	272.62	273.76	176.37	188.79	278.31	273.2 (188.8–278.3)

**Fig. 3:**
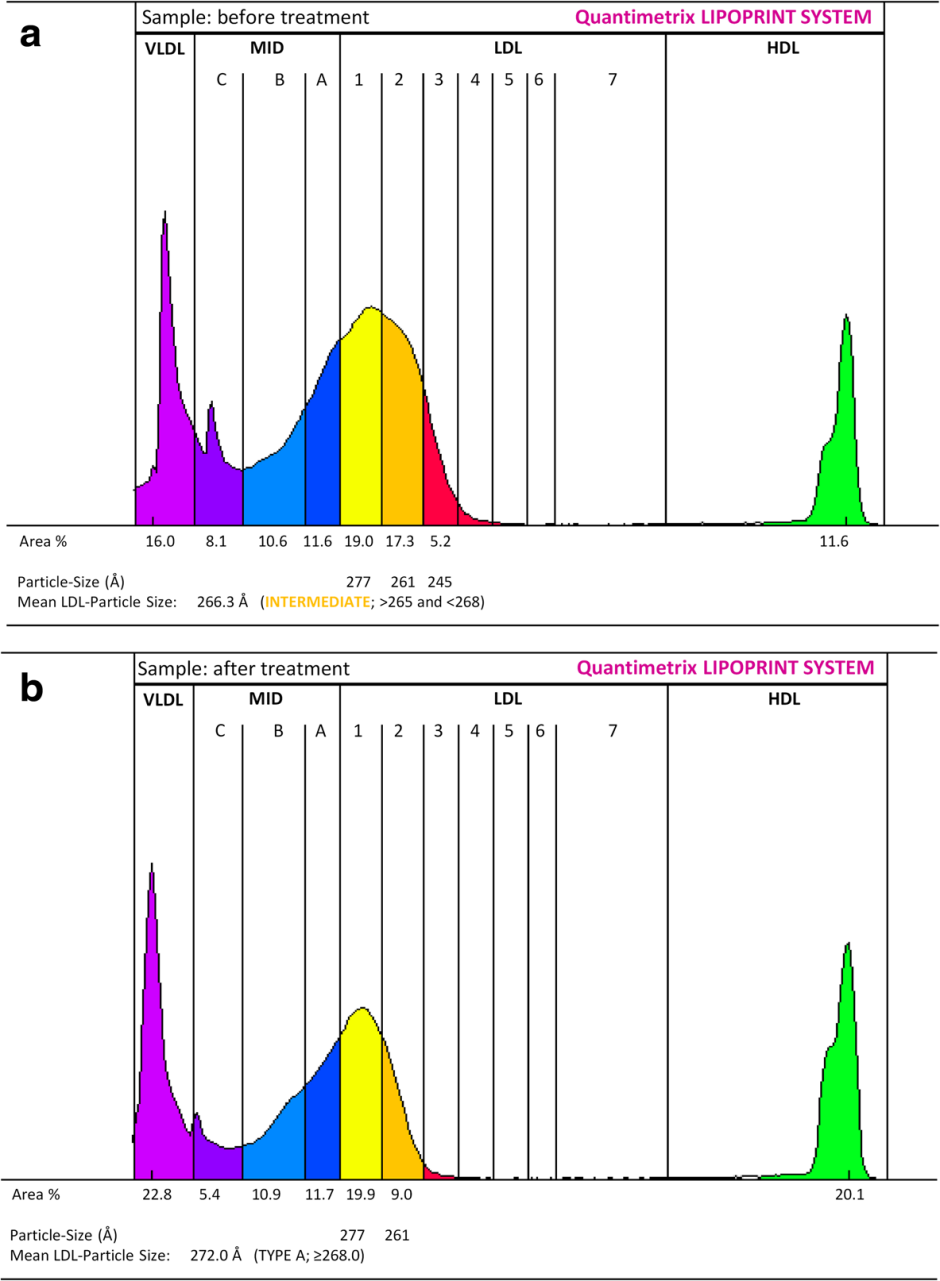
Representative lipoprotein subfraction patterns, before (**a**) and (**b**) after LDL apheresis in a patient with familial hypercholesterolemia. Small dense LDL subfractions are shown in red

Using Lipoprint, plasma LDL and HDL subfractions of selected cases were also measured before and after LDL apheresis sessions. After treatment, favorable quantitative and qualitative changes in LDL and HDL subfractions were observed (Table 1). Among these, LDL apheresis markedly reduced the small dense LDL subfractions (Table 1 and Fig. 3).

## Discussion

Concordant with previous data, selective LDL apheresis reduced LDL-C and Lp(a) levels in patients with FH. Additionally, LDL apheresis confers other pleiotropic effects protecting patients against atherosclerosis as it decreases the plasma concentrations of several pro-inflammatory markers including cytokines, chemokines, adhesion factors and C-reactive protein, too [4]. Although the mechanisms of these changes are still unknown, both direct binding and indirect changes via altered gene transcription and translation are suggested. Dihazi et al. compared serum samples by proteomic analysis before and after LDL apheresis [17] and they found significant depletion of more than 70 functional proteins including peptides that were involved in the coagulation system and that with adhesive, rheological and inflammatory actions. They concluded that there was a strong interaction between the column and serum proteins during treatment, while others found altered gene expression of various molecules after the apheresis [18–20].

To the best of our knowledge, the impact of LDL apheresis on serum chemerin level has not been investigated yet. In our study, we found that serum chemerin levels decreased markedly after selective LDL apheresis in FH patients. We also demonstrated – although only in one patient - that continuous treatment was necessary to maintain lower serum chemerin levels. Besides decreasing LDL-C levels, lowering the circulating chemerin concentrations may be an additional beneficial effect of LDL apheresis, due to the suggested pro-inflammatory and pro-atherogenic properties of chemerin. Since LDL apheresis is currently not a widely used method in the treatment of severe hyperlipidemias, we had a limited patient number. Therefore, further studies on larger patient populations are needed to prove the short- and long-term effects of selective LDL apheresis on serum chemerin levels.

After the treatment sessions, we could elute a significant amount of chemerin from the apheresis column; supporting the data that chemerin might physically bind to the apheresis column; however the exact mechanism of this binding is unknown. Although there is no information in the literature about the charge of chemerin, one may speculate that it could bind to the column directly, similarly to the positively charged ApoB100-containing lipoprotein particles. On the other hand, it is also possible that chemerin might be associated to the positively charged lipoprotein particles and therefore it binds indirectly to the adsorber column. Further studies are needed to explore the exact mechanism.

Previous studies demonstrated that LDL apheresis results in a decrease of the small dense, particularly pro-atherogenic LDL subfraction and it also modifies subfraction pattern into a more anti-atherogenic manner [21, 22]. In our study, we found that LDL subfractions changed favorably both in terms of quantity and proportions after apheresis, yielding a marked reduction in the small dense and highly atherogenic LDL subfractions. These subfractions possess an increased susceptibility for oxidative modification; therefore play a key role in the initiation and progression of atherosclerosis. Hence, reduction of these subfractions is of major importance in this high-risk population. Based upon our results, the efficacy of the treatment on lipid subfractions may also correlate with its chemerin lowering effect.

## Conclusions

To date, this is the first, although pilot, study on the impact of LDL apheresis on circulating chemerin levels. We also demonstrated that the chemerin lowering effect can be explained by the binding of the protein to the adsorber column during LDL apheresis. Further studies are needed to clarify the associations between the changes in the serum chemerin levels and the impact of LDL apheresis on lipid subfractions and their long-term associations with the atherosclerotic process. The better understanding of the pleiotropic, non-lipid effects of LDL apheresis may alter our therapeutic strategy in patients with severe hypercholesterolemia and may contribute to maximize the cardiovascular risk reduction in this patient population.

## Abbreviations

ACD-A: Acid citrate dextrose formula A
Apo: Apolipoprotein
AUC: Area under the curve
DALI: Direct adsorption of lipoproteins
IDL: Intermediate-density lipoprotein
FH: Familial hypercholesterolemia
HDL: High-density lipoprotein
HDL-C: HDL-cholesterol
LDL: Low-density lipoprotein
LDL-C: LDL-cholesterol
Lp(a): Lipoprotein(a)
PCSK9: Proprotein convertase subtilisin/kexin type 9
SPSS: Statistical Package for the Social Sciences
VLDL: Very-low-density lipoprotein
